# A physician’s guide to the 2-dose schedule of MenB-FHbp vaccine

**DOI:** 10.1080/21645515.2019.1596711

**Published:** 2019-05-22

**Authors:** Angee McDaniel, Amanda Dempsey, Amit Srivastava

**Affiliations:** a Medical Development, Scientific & Clinical Affairs, Pfizer Vaccines, Pfizer Inc, Collegeville, PA, USA; b University of Colorado Denver, Anschutz Medical Campus, Denver, CO, USA; c Medical Development, Scientific & Clinical Affairs, Pfizer Vaccines, Pfizer Inc, Cambridge, MA, USA

**Keywords:** Meningococcal disease, MenB-FHbp, vaccination, 2-dose, adolescents, meningococcal serogroup B, broad coverage, persistence, booster response

## Abstract

Meningococcal serogroup B (MenB) is the predominant cause of invasive meningococcal disease in the United States, with older adolescents and young adults attending college at increased risk. Notably, MenB caused all meningococcal disease outbreaks at US colleges between 2011 and 2018. MenB disease is vaccine-preventable. The MenB-FHbp vaccine can be administered on a 2-dose (0 and 6 months) schedule to healthy adolescents and young adults or as a tailored 3-dose (0, 1–2, and 6 months) schedule for individuals at increased risk. This review focuses on the 2-dose schedule (0 and 6 months) of MenB-FHbp. Clinical evidence demonstrating strong and broadly protective immunogenicity in adolescents after primary vaccination, immune persistence up to 48 months post-primary vaccination (18–61% of subjects across schedules), and immune memory evidenced by robust response to a single booster dose are described. Implementation approaches to ensure adolescents and young adults are fully vaccinated against meningococcal disease are discussed.

## Introduction

Invasive meningococcal disease (IMD) is a vaccine-preventable bacterial infection.^1^^,2^ Meningococcal serogroup B (MenB) is currently the predominant IMD-causing serogroup among US adolescents and young adults, with college students at increased risk.^3,4^ This review focuses on the more recently approved 2-dose schedule (administered at 0 and 6 months) of MenB-FHbp (Trumenba®; Pfizer Inc, Philadelphia, PA, USA), a MenB vaccine for healthy adolescents and young adults.^5^ The clinical data supporting breadth of immune coverage of circulating pathogenic MenB strains, short- and long-term immunogenicity, and establishment of immune memory evidenced by booster responses are described. Implementation approaches to ensure that adolescents and young adults are fully vaccinated against meningococcal disease are also discussed.

## Meningococcal disease epidemiology

IMD, caused by the Gram-negative bacterium *Neisseria meningitidis*, is an uncommon but serious infection that progresses rapidly such that within 24 h, the clinical manifestations can progress from flu-like symptoms to septicemia and/or meningitis.^1,6,7^ The nonspecific nature of early symptoms can often result in misdiagnosis or delay in treatment.^7^ Even with appropriate medical care, meningococcal disease is fatal in approximately 8–15% of cases and up to 20% of survivors experience debilitating long-term physical and/or neurologic effects, such as limb amputation, hearing loss/impairment, and pain.^6–8^

Of the six serogroups known to cause meningococcal disease globally (A, B, C, W, X, and Y),^8,9^ MenB is the leading cause of meningococcal disease in the United States.^3^ According to data from the Centers for Disease Control and Prevention (CDC) Enhanced Meningococcal Disease Surveillance (EMDS), the incidence of meningococcal disease is highest in infants younger than 1 y (0.63 per 100,000 individuals), with a secondary peak occurring among 16- to 23-y-olds (0.20 per 100,000 individuals). In both groups, MenB is the leading cause of disease, accounting for 60% and 69% of cases, respectively.^10^ MenB also has been the predominant cause of death due to meningococcal disease across all ages in the United States in 2015 and 2016 and the second leading cause of death in 2017 (deaths per year for 2015, 2016, and 2017 were 17, 14, and 16 for serogroup B; 8, 12, and 18 for serogroup C; 5, 6, and 3 for serogroup W; 10, 9, and 2 for serogroup Y; and 11, 7, and 6 for ungroupable/unknown, respectively).^3,10,11^

Meningococcal carriage peaks at approximately 19 y of age^12^ and is a precondition for transmission and invasive disease.^8,12^ In this epidemiologic context of heightened carriage and IMD among older adolescents, although the incidence is low, US college students are demonstrably at increased risk for MenB disease. Specifically, EMDS data from 2014 to 2016 revealed that 18- to 24-y-olds attending college have a threefold higher risk for MenB disease compared with those not attending college (relative risk [RR], 3.54 [95% confidence interval (CI), 2.21–5.41]); in contrast, there was no difference in the combined risk of disease caused by serogroups C, W, and Y between college students and those not attending college (RR, 0.56 [95% CI, 0.27–1 .14]).^13^ MenB has caused all US college outbreaks of meningococcal disease from 2011 to 2018.^14–17^ Even after excluding campus outbreak cases, college students’ risk of disease remains elevated (RR, 2.76 [95% CI, 1.73–4.44]),^13^ although the majority (~68%) of MenB cases occur sporadically among 18- to 24-y-olds and are not associated with outbreaks.^13^

## Meningococcal vaccines and Advisory Committee on Immunization Practices recommendations

Two classes of vaccines help protect against meningococcal disease: quadrivalent conjugate MenACWY vaccines, which are based on capsular polysaccharide A, C, W, and Y antigens coupled to carrier proteins,^18–20^ and MenB vaccines, which are based on subcapsular protein antigens.^5,21^

Quadrivalent MenACWY vaccines have been licensed in the United States since 2005^1^ and are universally recommended (Category A^22^) by the US Advisory Committee on Immunization Practices (ACIP) for all 11- to 12-y-olds, with a booster dose at 16 y of age (Table 1).^1^ Two available MenACWY vaccines are MenACWY-CRM (Menveo®, GSK Vaccines Srl, Sovicille, Italy) and MenACWY-D (Menactra®, Sanofi Pasteur Inc., Swiftwater, PA, USA).^18,19^

**Table 1.** Summary of ACIP recommendations for meningococcal vaccination and dosing of adolescents.

Vaccine	Target group	Primary dosing	Booster dose	Category^a^
MenB^2,23,25^	Healthy individuals aged 16–23 y (16–18 y preferred)	2 doses	–	B
	Individuals aged ≥10 y at risk due to Persistent complement component deficiencies Anatomic or functional asplenia Routine exposure as a microbiologist Serogroup B meningococcal disease outbreak	2 or 3 doses (vaccine-dependent)	–	A
MenACWY^1^	Healthy individuals aged 11–18 y (11–12 y preferred)^b^	1 dose	1 dose, if primary dosing given before age 16 y	A
	Individuals with Persistent complement deficiencies Functional or anatomic asplenia	2 doses	1 dose 5 y after primary dosing and repeated every 5 y thereafter	A
	Individuals who are First-year college students aged ≤21 y and living in residential housing Traveling to or residents of countries where meningococcal disease is epidemic or hyper-endemic At risk during an outbreak attributed to a vaccine serogroup Are microbiologists routinely exposed to *Neisseria meningitidis*	1 dose	1 dose 5 y after primary dosing and repeated every 5 y thereafter^b^	A

ACIP = Advisory Committee on Immunization Practices; MenACWY = meningococcal serogroups A, C, W, and Y vaccine; MenB = meningococcal serogroup B vaccine. ^a^ACIP Category A represents a recommendation for all those in an age- or risk factor-based group; Category B is a recommendation for individual clinical decision-making. ^b^For individuals who remain at increased risk.

**Figure 1.**
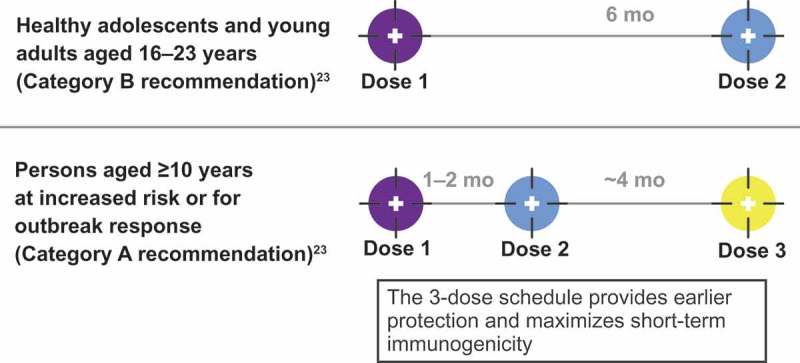
US Advisory Committee on Immunization Practices recommendations for MenB-FHbp 2-dose and 3-dose schedules.^23^ For the 2-dose schedule: if dose 2 is administered <6 months after dose 1, a third dose should be given ≥4 months after dose 2. For the 3-dose schedule: if dose 2 is administered ≥6 months after dose 1, a third dose does not need to be administered.

MenB vaccines were first licensed in the United States in 2014 under the US Food and Drug Administration (FDA) accelerated approval process in response to two US college-based MenB outbreaks in 2013.^2^ Two products are currently approved for use in adolescents and young adults 10–25 y of age: MenB-FHbp and MenB-4C (Bexsero®; GlaxoSmithKline Vaccines, Srl, Siena, Italy).^5,21^ MenB-FHbp is composed of two recombinant lipidated FHbp variants, one each from subfamily A and subfamily B.^5^ MenB-FHbp is administered in either a 2-dose schedule (at 0 and 6 months) to healthy individuals who are not at increased disease risk or a tailored 3-dose schedule (at 0, 1–2, and 6 months) for those at increased risk of meningococcal disease and in response to MenB disease outbreaks, to provide earlier protection and maximize short-term immunogenicity (Figure 1).^23^ MenB-4C is composed of recombinant proteins: neisserial adhesin A (NadA), neisserial heparin-binding antigen (NHBA), non-lipidated FHbp subfamily B, and outer membrane vesicles (OMVs) containing PorA P1.4 (VR2).^21,24^ MenB-4C is administered in a 2-dose schedule with at least 1 month between doses.^21^ Because the two MenB vaccines are compositionally distinct and have different immunization schedules, they are not interchangeable and ACIP does not state a preference; once a vaccination series is started, the same product must be used to complete the series.^23^ MenB vaccines have an ACIP Category A recommendation for individuals ≥10 y of age who are at increased risk for MenB disease, such as those with asplenia, complement deficiency, or laboratory or outbreak exposure,^2^ and a Category B recommendation (individual clinical decision-making in consultation with a health-care provider) for healthy individuals 16–23 y of age (16–18 y preferred)^23,25^ (Table 1); currently, there are no recommendations regarding booster dosing with MenB vaccines.

## How was the breadth of MenB-FHbp coverage evaluated?

Vaccine-elicited protection against meningococcal infection is evaluated by the *in vitro* serum bactericidal assay using human complement (hSBA), which measures antibody and complement-mediated killing of meningococcal test strains.^26^ An hSBA titer (i.e., the highest serum dilution that kills ≥50% of test bacteria^26^) of ≥1:4 is widely accepted by the US FDA and other regulators as a correlate of protection against IMD,^27–30^ and has been used for licensure of MenACWY, MenB, and other meningococcal vaccines.^5,18,19,21^ The hSBA titer for protein-based vaccines is influenced by antigen sequence variation and surface protein expression level in circulating meningococcal strains.^26^ Therefore, choice of test strains for use in hSBAs to determine breadth of coverage is critical for MenB vaccines.

FHbp is a surface-exposed protein present on nearly all meningococcal strains that consists of two distinct subfamilies (A and B);^31^ the bivalent MenB-FHbp vaccine contains both FHbp-A and B proteins.^32^ Furthermore, >91% of invasive MenB strains express FHbp at sufficient levels to be susceptible to MenB-FHbp-elicited bactericidal antibodies.^33^ MenB-FHbp-elicited protective responses were evaluated in clinical studies by measuring hSBA responses against a diverse panel of four primary MenB test strains (A22 and A56 from FHbp subfamily A and B24 and B44 from FHbp subfamily B) and ten additional strains. These 14 test strains differ from the vaccine antigens and collectively represent >83% epidemiologic coverage of circulating disease strains in the United States.^34^ In pivotal phase 3 studies, responses to the four primary strains predicted responses to the ten additional strains.^32^

## What is the clinical evidence supporting the 2-dose series of MenB-FHbp?

The immunogenicity and safety of MenB-FHbp has thus far been evaluated in 11 published clinical studies wherein more than 15,000 adolescents and young adults received the vaccine.^35^ These studies evaluated 2- and 3-dose schedules, long-term immunogenicity, booster dose responses, and co-administration with vaccines commonly given to adolescents, including MenACWY-D, Tdap (Adacel®, Sanofi Pasteur Inc., Swiftwater, PA, USA), 4vHPV (Gardasil®, Merck & Co, Inc., Whitehouse Station, NJ, USA), and Tdap/IPV (Repevax®, Sanofi Pasteur, Lyon, France) vaccines.^36–38^

**Figure 2.**
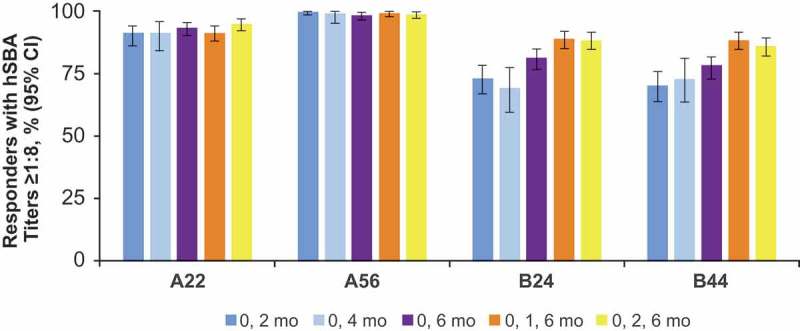
Clinical data supporting MenB-FHbp 2- and 3-dose schedules to elicit protective responses against diverse primary strain panels.^39^ Percentage of subjects demonstrating a protective hSBA titer of ≥1:8 against a diverse panel of test strains is shown from sera collected 1 month after the final dose (after the second dose for the 2-dose schedules [0 and 2 months, 0 and 4 months, or 0 and 6 months] and after the third dose for the 3-dose schedules [0, 1, and 6 months or 0, 2, and 6 months]). hSBA = serum bactericidal assay using human complement.

Clinical data supporting the MenB-FHbp 2-dose schedule were from a phase 2 randomized clinical study in healthy adolescents aged 11–18 y that evaluated multiple schedules: 2 doses (administered at 0 and 2 months, 0 and 4 months, or 0 and 6 months) or 3 doses (at 0, 1, and 6 months or 0, 2, and 6 months).^39^ An hSBA titer ≥1:8, which is more stringent than the recognized correlate of protection of ≥1:4,^27–29^ was used as the primary end point of the study.^39^ Before vaccination, 4.4–28.1% of subjects had hSBA titers ≥1:8.^40^ Upon completion of a 2-dose MenB-FHbp series, broadly protective responses were elicited in 70.1–100% of subjects against the diverse panel of four primary strains (Figure 2).^39^ In the same study, similar response rates (86.1–99.4%) were observed for both of the 3-dose series. For all assessed dosing regimens in this study, statistically significant increases in hSBA titers ≥1:8 were observed from before vaccination to 1 month following completion of the MenB-FHbp series. In addition, similar percentages of subjects achieved greater than or equal to fourfold increases in hSBA titers over baseline at 1 month after completion of the vaccination series: 64.5–90.1% for the 2-dose series and 74.1–93.8% for the 3-dose series.^5^ Importantly, the composite response for all four primary strains combined was similar for the 2-dose schedule (0 and 6 months; 72.9% [95% CI, 65.9–79.1]) and 3-dose schedule (0, 1 and 6 months; 80.3% [95% CI, 73.7–85.9]; 0, 2 and 6 months; 81.8% [95% CI, 74.9–87.4]) at 1 month after completion of the primary vaccination series.^5^ Reports of adverse events were similar across the dosing schedules tested, were mostly mild or moderate in severity, and did not potentiate with subsequent doses.^39^ Notably, immunogenicity of the 2-dose series generally increased with the length of interval between the 2 doses and was highest in those receiving vaccine at 0 and 6 months (Figure 2).^39^

**Figure 3.**
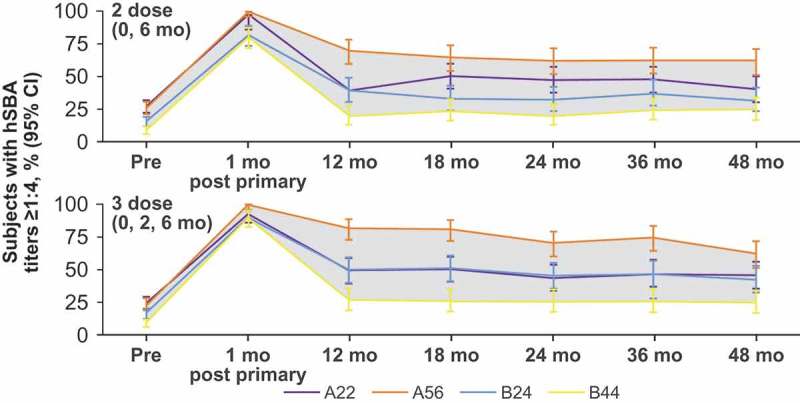
Persistence of hSBA responses up to 48 months after primary vaccination against the four primary test strains for the 2-dose (0 and 6 months) and 3-dose (0, 2, and 6 months) MenB-FHbp schedules.^23^ Because the 4-strain test panel is epidemiologically representative of diverse, circulating disease-causing strains,^34^ the shaded area between the curves represents the range of subjects with protective immune responses against most circulating MenB strains. hSBA = serum bactericidal assay using human complement.

Vaccine-elicited immune responses peak after vaccination and then progressively wane with time.^41^ Therefore, the long-term immunogenicity of MenB-FHbp was evaluated over a period of 4 y after primary vaccination with either the 2-dose or 3-dose schedule, followed by a single booster dose to evaluate immune memory; hSBA responses against the same diverse panel of four primary strains were measured.^23^ Regardless of the dosing schedule used, the proportion of MenB-FHbp recipients with protective antibodies (hSBA titer ≥1:4) across primary test strains and dose schedules peaked 1 month after primary vaccination (78.9–99.1% of subjects^23^) then declined over the course of 12 months (16.5–76.1% of subjects achieved hSBA titers ≥1:8 or 1:16 at 12 months^42^) and subsequently remained stable for up to 48 months (18.0–61.3% of subjects achieved hSBA titers ≥1:8 or 1:16 at 48 months^42^) (Figure 3).^23^ After administration of a single MenB-FHbp booster dose approximately 48 months after the primary series, hSBA response rates met or exceeded those at 1 month after the primary series, exemplifying a strong and broadly protective anamnestic response.^23^ Data on the long-term persistence of the antibody response to the MenB-FHbp booster dose are not yet published.

## What is the clinical evidence for the protective response of MenB-FHbp and MenB-4c against antigenically diverse circulating strains?

Antibody response to MenB vaccines is affected by the degree of match between the vaccine antigens and circulating pathogenic strains, immunogenicity of vaccine antigen(s), and immune responses of the vaccinated individual. The sub-capsular proteins in MenB vaccines have multiple sequence variants and have differing surface expression levels on circulating pathogenic strains,^26^ both of which affect the bactericidal activity of vaccine-elicited antibodies. Therefore, to demonstrate broad protective coverage while targeting an antigen that has variability, it is essential to evaluate each MenB vaccine by hSBA against a panel of diverse strains that differ from the vaccine antigen and are epidemiologically representative (i.e., outbreak and non-outbreak/endemic strains).

**Figure 4.**
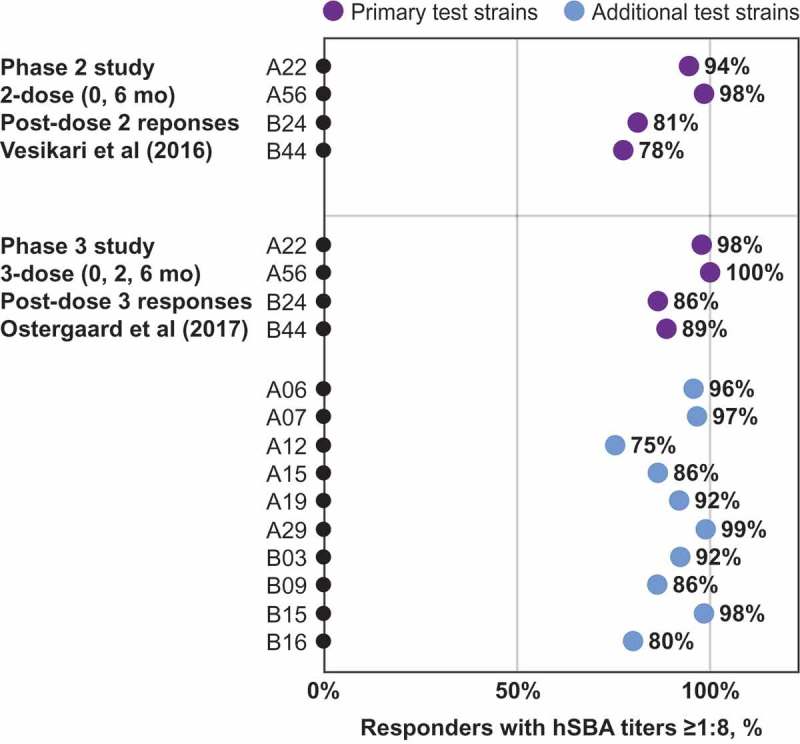
MenB-FHbp shows consistent protective responses across diverse MenB strains – including FHbp subfamilies A and B. Point estimates from two clinical studies showing the percentage of MenB-FHbp recipients demonstrating a protective hSBA titer ≥1:8 against a diverse panels of test strains after vaccination with the MenB-FHbp 2-dose (0 and 6 months)^39,40^ or 3-dose (0, 2, and 6 months) schedule.^32^ The primary test panel of four strains (two from FHbp subfamily A and two from FHbp subfamily B was evaluated in both phase 2 and phase 3 studies shown; a panel of ten additional test strains (six from FHbp subfamily A and four from FHbp subfamily B was evaluated in the phase 3 study with the 3-dose schedule. For the phase 3 study, data in adolescents are shown.^32^

**Figure 5.**
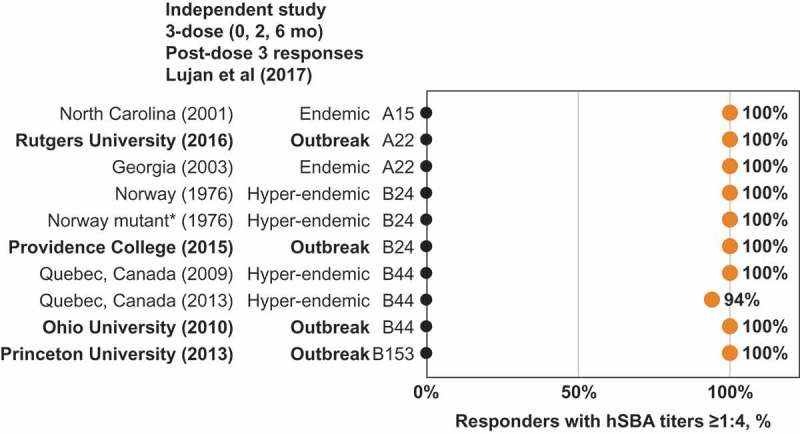
MenB-FHbp shows consistent protective responses across diverse MenB strains – including FHbp subfamilies A and B and outbreak strains. Point estimates from an independent clinical study showing the percentage of MenB-FHbp recipients demonstrating a protective hSBA titer of ≥1:4 against a diverse panel of test strains (three FHbp subfamily A and seven FHbp subfamily B strains) after vaccination with MenB-FHbp 3-dose (0, 2, and 6 months) series.^46^ The full test panel (six FHbp subfamily A and eight FHbp subfamily B strains) included endemic and hyper-endemic MenB disease-causing strains, and strains from campus outbreaks in the United States; a subset of that test panel, for which percentages of subjects showing immune titers ≥1:4 were reported in the publication, is shown. *Mutant Norway 1976 strain expresses 50% less FHbp subfamily B. FHbp = factor H binding protein; hSBA = serum bactericidal assay using human complement.

MenB-FHbp demonstrated consistent responses against diverse panels of test strains with 2-dose and 3-dose schedules as seen in the phase 2 and phase 3 studies of the clinical development program (Figure 4).^32,39^ Among adolescents immunized with a 3-dose MenB-FHbp series, additional clinical studies have reported similar protective responses after 2 or 3 doses, including a larger panel of 27 diverse MenB strains containing isolates from two US college outbreaks, as well as a panel of six French outbreak strains.^43–46^ An independent clinical study of 17 laboratory and health-care workers immunized with a 3-dose MenB-FHbp series evaluated hSBA activity (titer ≥1:4) against a large and diverse panel of endemic and hyper-endemic MenB disease-causing strains and strains from six US college outbreaks; this study found protective responses consistent with those seen within the MenB-FHbp clinical program (Figure 5 shows a subset of these strains, including four from US college outbreaks).^46^ Taken together, these data provide continued confidence that the MenB-FHbp vaccine 2-dose regimen provides sufficient broad and sustained protective coverage against the diversity of circulating MenB strains in comparison with the 3-dose regimen.

The MenB-4C vaccine contains NHBA, FHbp-subfamily B, NadA, and OMV (containing PorA P1.4 [VR2]).^21,24^ These protein antigens are present in approximately 98%, 60%, 30%, and 5.3%, respectively, of disease-causing MenB strains in the United States based on evaluation of gene presence and protein expression in a panel of US invasive clinical isolates grown *in vitro* at the Bacterial Meningitis Laboratory of the CDC.^47,48^ MenB-4C clinical studies for licensure evaluated hSBA responses with closely matched, antigen-specific (vaccine homologous) indicator strains rather than strain panels, for three of the four antigens (no strain was evaluated for the NHBA antigen for US licensure).^49^ Two clinical studies reported that after completing the 2-dose schedule, protective pre- and post-vaccination responses increased from 2–7% to 70–100% among 11- to 17-y-old subjects^50^ and from 4–5% to 82–93% among 10- to 25-y-old subjects (an hSBA titer ≥1:5 was used, which is more stringent than the accepted immune correlate of 1:4).^51^ Two additional studies reported that after completing the 2-dose schedule, pre- and post-vaccination responses increased from 34–44% to >99% among 11- to 17-y-olds^52^ and from 57–69% to 99–100% among 18- to 24-y-old subjects (hSBA titer ≥1:4).^53^ An independent, real-world study examined pre- and post-vaccination immune responses to the MenB-4C 2-dose schedule (administered at 0 and 1 month) deployed in the 2013–2014 Princeton University MenB outbreak.^54^ This study showed that 83–98% of those who received MenB-4C seroconverted against two antigen-specific indicator strains (greater than or equal to fourfold rise in hSBA titer) and 20% of MenB-4C recipients seroconverted against the actual MenB outbreak strain.^55^ Lack of hSBA data against MenB strain panels makes it difficult to assess breadth of immune coverage by MenB-4C, though vaccine effectiveness data among infants (82.9%; 95% CI, 24.1–95.2) have been reported from the first 10 months of the United Kingdom’s national immunization program.^56^

## What are known barriers to adolescent immunization delivery and what are potential solutions to these barriers?

The adolescent population is difficult to reach for immunization: they are generally healthy and tend to have fewer medical visits as they move through their teens, with a decline in number of visits at around 16 y of age.^57^ According to the 2014 Medical Expenditure Panel Survey, only 54% of those aged 9–21 y saw a primary care physician,^57^ which is likely the care provider from whom the majority of adolescents receive vaccines. Individuals in this age group are also transitioning between providers: pediatricians cared for the majority of 11- to 17-y-olds and family practitioners saw most 18- to 21-y-olds.^57^ Parents expect their children’s physicians to provide guidance on adolescent vaccinations,^58^ but studies have shown that these physicians struggle to understand the reasons for different types of meningococcal vaccines and the ACIP meningococcal vaccine recommendations.^59^ For MenB vaccines, the ACIP Category B recommendation requires individual clinical decision-making in the context of a patient-provider discussion,^60^ and several studies have shown that this recommendation is especially confusing for health-care providers to implement.^59,61–63^ However, it is noteworthy that the American Academy of Pediatrics states that not discussing MenB disease and the available licensed vaccines with families is not an option.^60^ Taken together, these factors likely contribute to the disparate immunization rates for adolescent vaccines. According to the most recent (2017) National Immunization Survey–Teen data, the immunization rate for multidose MenB vaccines, collected 2 y after the initial (2015) recommendations for vaccinating healthy persons 16–23 y of age (16–18 y preferred), was approximately 14.5% for ≥1 dose among 17-y-olds.^64^ For context, the vaccination rate for the primary MenACWY dose (at 11–12 y^65^) is 84% among 13-y-olds, but the rate for the booster dose (at 16 y^65^) is only 44% among 17-y-olds.^64^ In addition, 48.6% of adolescents are now up-to-date with the human papilloma virus (HPV) 2-dose vaccination series (at 11–12 y of age).^64^ Single-dose Tdap^65^ remains the benchmark, with an 88.7% vaccination rate for ≥1 dose among adolescents 13–17 y of age. It is notable that MenB vaccines are comparatively newer, have a multidose regimen, and have a non-routine ACIP recommendation for a population that is known to be a challenge to vaccinate, all of which can affect immunization rates.

Age-based immunization platforms are catalysts for vaccination because they provide a forum for discussion and create an expectation of vaccination during the medical visit. The CDC’s nascent immunization platform for 16-y-olds is an essential tool for improving adolescent immunization delivery, akin to the successful immunization platform for 11-y-olds that was created in 1996 and has helped improve adolescent vaccination rates substantially.^66^ An immunization platform for 16-y-olds is strongly supported by the Society for Adolescent Health and Medicine, the Immunization Action Coalition, and the Adolescent Immunization Initiative.^66–68^ In addition, the 2017–2018 CDC Childhood Immunization Schedule newly highlights the immunization platform at 16 y of age as a shaded column to increase awareness among providers and parents that two meningococcal vaccines (MenACWY and MenB) are recommended for that age group.^65,69^ The MenB-FHbp 0- and 6-month schedule is comparable to other vaccines such as HPV and hepatitis A that are also given on a 0- and 6-month schedule and are familiar to providers,^65^ and a 2-dose series (rather than 3-dose) can potentially help improve adherence and series completion. Opportunities for improving adolescent vaccination rates include discussing vaccination at sports and camp physicals, during annual administration of flu vaccines, at acute care and follow-up visits, and during routine visits for chronic illness.^67^ In addition, reminding parents and providers that all ACIP-recommended vaccinations (Category A or B) are covered currently under the Affordable Care Act would be helpful.^60^

## Conclusion

Vaccination remains the best means of preventing MenB disease, which is currently the predominant IMD-causing serogroup among US adolescents and young adults; college students are especially at increased risk. Vaccination with both MenACWY and MenB is needed for complete protection against the most common serogroups causing meningococcal disease. For healthy adolescents, the licensed MenB-FHbp 2-dose regimen (0 and 6 months) was deemed to provide sufficient protection in comparison with the 3-dose regimen, is recommended by ACIP, and can be conveniently administered as part of the CDC’s 16-y-old immunization platform along with the MenACWY booster dose. For those at increased risk congenitally (e.g., due to asplenia or complement deficiency) or situationally (e.g., due to outbreaks), the tailored 3-dose regimen (0, 1–2, and 6 months) of MenB-FHbp provides rapid protection and maximizes short-term immunogenicity. As summarized herein, substantial clinical data are available on the breadth and duration of broad immunity elicited by the MenB-FHbp 2-dose series against genetically diverse MenB disease-causing strains and on the concomitant use of MenB-FHbp with other adolescent vaccines.

## References

[1] Cohn AC, MacNeil JR, Clark TA, Ortega-Sanchez IR, Briere EZ, Meissner HC, Baker CJ, Messonnier NE, Centers for Disease Control and Prevention Prevention and control of meningococcal disease: recommendations of the Advisory Committee on Immunization Practices (ACIP). MMWR Recomm Rep. 2013;62(RR–2):1–28. pii: rr6202a1. 23515099

[2] Folaranmi T, Rubin L, Martin SW, Patel M, MacNeil JR. Use of serogroup B meningococcal vaccines in persons aged ≥10 years at increased risk for serogroup B meningococcal disease: recommendations of the Advisory Committee on Immunization Practices, 2015. MMWR Morb Mortal Wkly Rep. 2015;64:608–12. 26068564 PMC4584923

[3] Centers for Disease Control and Prevention Enhanced meningococcal disease surveillance report, 2016 [accessed 2018 926]. https://www.cdc.gov/meningococcal/downloads/NCIRD-EMS-Report.pdf.

[4] Mbaeyi SA, Joseph SJ, Blain A, Wang X, Hariri S, MacNeil JR. Meningococcal disease among college-aged young adults: 2014-2016. Pediatrics. 2019;143(1):e20182130. doi:10.1542/peds.2018-2130. 30598460

[5] Trumenba® (meningococcal group B vaccine) Full prescribing information. Philadelphia (PA): Wyeth Pharmaceuticals Inc., a subsidiary of Pfizer Inc; 2017.

[6] Pace D, Pollard AJ Meningococcal disease: clinical presentation and sequelae. Vaccine. 2012;30(suppl 2):B3–B9. doi:10.1016/j.vaccine.2011.12.062. 22607896

[7] Martinón-Torres F Deciphering the burden of meningococcal disease: conventional and under-recognized elements. J Adolesc Health. 2016;59(2 suppl):S12–20. doi:10.1016/j.jadohealth.2016.03.041. 27449145

[8] World Health Organization Meningococcal meningitis. [accessed 2018 926]. http://www.who.int/immunization/diseases/meningitis/en/.

[9] Harrison LH, Trotter CL, Ramsay ME Global epidemiology of meningococcal disease. Vaccine. 2009;27(suppl 2):B51–63. doi:10.1016/j.vaccine.2009.04.063. 19477562

[10] Centers for Disease Control and Prevention Enhanced meningococcal disease surveillance report, 2017 [accessed 2018 1130]. https://www.cdc.gov/meningococcal/downloads/NCIRD-EMS-Report-2017.pdf.

[11] Centers for Disease Control and Prevention Enhanced meningococcal disease surveillance report, 2015 [accessed 2018 619]. https://www.cdc.gov/meningococcal/downloads/NCIRD-EMS-Report-2015.pdf.

[12] Christensen H, May M, Bowen L, Hickman M, Trotter CL Meningococcal carriage by age: a systematic review and meta-analysis. Lancet Infect Dis. 2010;10(12):853–61. doi:10.1016/S1473-3099(10)70251-6. 21075057

[13] Meyer S Epidemiology of meningococcal disease among college students – United States, 2014-2016 [accessed 2018 42]. https://www.cdc.gov/vaccines/acip/meetings/downloads/slides-2018-02/Mening-02-Meyer-508.pdf.

[14] Biswas HH, Han GS, Wendorf K, Winter K, Zipprich J, Perti T, Martinez L, Arellano A, Kyle JL, Zhang P, et al Notes from the field: outbreak of serogroup B meningococcal disease at a university - California, 2016. MMWR Morb Mortal Wkly Rep. 2016;65(20):520–21. doi:10.15585/mmwr.mm6520a3. 27227576

[15] Meyer S Update on the epidemiology of meningococcal disease and guidance for the control of meningococcal disease outbreaks in the U.S. [accessed 2018 926]. https://pdfs.semanticscholar.org/presentation/e0b3/aa069eb91e1d831b8c40d4340d21afa4c513.pdf.

[16] De Maria A Update: invasive meningococcal cases at the University of Massachusetts (UMass) Amherst. Jamaica Plain (MA): Commonwealth of Massachusetts Department of Public Health, editor; 2017.

[17] California Health Alert Network San Diego Invasive meningococcal disease outbreak at San Diego State University; County of San Diego: San Diego, CA 2018.

[18] Menactra® (meningococcal [groups A, C, Y and W-135] polysaccharide diphtheria toxoid conjugate vaccine) Full prescribing information. Swiftwater (PA): Sanofi Pasteur Inc; 2016.

[19] Menveo® (meningococcal [groups A, C, Y and W-135] oligosaccharide diphtheria CRM_197_ conjugate vaccine) Full prescribing information. Sovicille (Italy): GSK Vaccines S.r.l; 2017.

[20] NIMENRIX (meningococcal polysaccharide serogroups A, C, W-135 and Y conjugate vaccine) Summary of product characteristics. Sandwich (Kent, UK): Pfizer Limited; 2017.

[21] Bexsero (MenB-4C) Summary of product characteristics. Siena (Italy): GlaxoSmithKline Vaccines Srl; 2018.

[22] MacNeil JR, Rubin LG, Patton M, Ortega-Sanchez IR, Martin SW Recommendations for use of meningococcal conjugate vaccines in HIV-infected persons — Advisory Committee on Immunization Practices, 2016. MMWR Morb Mortal Wkly Rep. 2016;65(43):1189–94. doi:10.15585/mmwr.mm6543a3. 27811836

[23] Patton ME, Stephens D, Moore K, MacNeil JR Updated recommendations for use of MenB-FHbp serogroup B meningococcal vaccine — advisory Committee on Immunization Practices, 2016. MMWR Morb Mortal Wkly Rep. 2017;66(19):509–13. doi:10.15585/mmwr.mm6619a6. 28520709 PMC5657641

[24] McNeil LK, Zagursky R, Shuo L, Murphy E, Zlotnick G, Hoiseth SK, Jansen KU, Andersen AS The role of factor H binding protein in *Neisseria meningitidis* virulence and its potential as a vaccine candidate to broadly protect against meningococcal disease. Microbiol Mol Biol Rev. 2013;77(2):234–52. doi:10.1128/MMBR.00056-12. 23699256 PMC3668674

[25] MacNeil JR, Rubin L, Folaranmi T, Ortega-Sanchez IR, Patel M, Martin SW Use of serogroup B meningococcal vaccines in adolescents and young adults: recommendations of the Advisory Committee on Immunization Practices, 2015. MMWR Morb Mortal Wkly Rep. 2015;64(41):1171–76. doi:10.15585/mmwr.mm6441a3. 26492381

[26] Donald RG, Hawkins JC, Hao L, Liberator P, Jones TR, Harris SL, Perez JL, Eiden JJ, Jansen KU, Anderson AS Meningococcal serogroup B vaccines: estimating breadth of coverage. Hum Vaccin Immunother. 2017;13(2):255–65. doi:10.1080/21645515.2017.1264750. 27960595 PMC5328210

[27] Goldschneider I, Gotschlich EC, Artenstein MS Human immunity to the meningococcus. I. The role of humoral antibodies. J Exp Med. 1969;129:1307–26. 4977280 10.1084/jem.129.6.1307 PMC2138650

[28] Borrow R, Aaberge IS, Santos GF, Eudey TL, Oster P, Glennie A, Findlow J, Hoiby EA, Rosenqvist E, Balmer P, et al Interlaboratory standardization of the measurement of serum bactericidal activity by using human complement against meningococcal serogroup b, strain 44/76-SL, before and after vaccination with the Norwegian MenBvac outer membrane vesicle vaccine. Clin Diagn Lab Immunol. 2005;12(8):970–76. doi:10.1128/CDLI.12.8.970-976.2005. 16085915 PMC1182195

[29] Andrews N, Borrow R, Miller E Validation of serological correlate of protection for meningococcal C conjugate vaccine by using efficacy estimates from postlicensure surveillance in England. Clin Diagn Lab Immunol. 2003;10(5):780–86. doi:10.1128/CDLI.10.5.780-786.2003. 12965904 PMC193909

[30] Borrow R, Carlone GM, Rosenstein N, Blake M, Feavers I, Martin D, Zollinger W, Robbins J, Aaberge I, Granoff DM, et al *Neisseria meningitidis* group B correlates of protection and assay standardization–international meeting report Emory University, Atlanta, Georgia, United States, 16-17 March 2005. Vaccine. 2006;24:5093–107. 16838413 10.1016/j.vaccine.2006.03.091

[31] Granoff DM, Pollard AJ, Harrison LH Meningococcal capsular group B vaccines In: Plotkin SA, Orenstein WA, Offit PA, Edwards KM editors. Plotkin’s Vaccines. Elsevier: Philadelphia, PA; 2018 p. 644–662.e646.

[32] Ostergaard L, Vesikari T, Absalon J, Beeslaar J, Ward BJ, Senders S, Eiden JJ, Jansen KU, Anderson AS, York LJ, et al A bivalent meningococcal B vaccine in adolescents and young adults. N Engl J Med. 2017;377(24):2349–62. doi:10.1056/NEJMoa1614474. 29236639

[33] McNeil LK, Donald RGK, Gribenko A, French R, Lambert N, Harris SL, Jones TR, Li S, Zlotnick G, Vogel U, et al Predicting the susceptibility of meningococcal serogroup B isolates to bactericidal antibodies elicited by bivalent rLP2086, a novel prophylactic vaccine. MBio. 2018;9(2):e00036–00018. doi:10.1128/mBio.00036-18. 29535195 PMC5850321

[34] European Medicines Agency Assessment report: Trumenba [accessed 2017 77]. http://www.ema.europa.eu/docs/en_GB/document_library/EPAR_-_Public_assessment_report/human/004051/WC500228997.pdf.

[35] Perez JL, Absalon J, Beeslaar J, Balmer P, Jansen KU, Jones TR, Harris S, York LJ, Jiang Q, Radley D, et al From research to licensure and beyond: clinical development of MenB-FHbp, a broadly protective meningococcal B vaccine. Expert Rev Vaccines. 2018;17(6):461–77. doi:10.1080/14760584.2018.1483726. 29883226

[36] Vesikari T, Wysocki J, Beeslaar J, Eiden J, Jiang Q, Jansen KU, Jones TR, Harris SL, O’Neill RE, York LJ, et al Immunogenicity, safety, and tolerability of bivalent rLP2086 meningococcal group B vaccine administered concomitantly with diphtheria, tetanus, and acellular pertussis and inactivated poliomyelitis vaccines to healthy adolescents. J Pediatric Infect Dis Soc. 2016;5(2):180–87. doi:10.1093/jpids/piv064. 26803328 PMC5407129

[37] Muse D, Christensen S, Bhuyan P, Absalon J, Eiden JJ, Jones TR, York LJ, Jansen KU, O’Neill RE, Harris SL, et al A phase 2, randomized, active-controlled, observer-blinded study to assess the immunogenicity, tolerability and safety of bivalent rLP2086, a meningococcal serogroup B vaccine, coadministered with tetanus, diphtheria and acellular pertussis vaccine and serogroup A, C, Y and W-135 meningococcal conjugate vaccine in healthy US adolescents. Pediatr Infect Dis J. 2016;35(6):673–82. doi:10.1097/INF.0000000000001124. 26974889

[38] Senders S, Bhuyan P, Jiang Q, Absalon J, Eiden J, Jones TR, York LJ, Jansen KU, O’Neill RE, Harris SL, et al Immunogenicity, tolerability, and safety in adolescents of bivalent rLP2086, a meningococcal serogroup B vaccine, coadministered with quadrivalent human papilloma virus vaccine. Pediatr Infect Dis J. 2016;35(5):548–54. doi:10.1097/INF.0000000000001072. 26835974

[39] Vesikari T, Ostergaard L, Diez-Domingo J, Wysocki J, Flodmark CE, Beeslaar J, Eiden J, Jiang Q, Jansen KU, Jones TR, et al Meningococcal serogroup B bivalent rLP2086 vaccine elicits broad and robust serum bactericidal responses in healthy adolescents. J Pediatric Infect Dis Soc. 2016;5(2):152–60. doi:10.1093/jpids/piv039. 26407272 PMC5407127

[40] Study B1971012 Data on File New York (NY): Pfizer Inc; 2013.

[41] Gu XX, Plotkin SA, Edwards KM, Sette A, Mills KHG, Levy O, Sant AJ, Mo A, Alexander W, Lu KT, et al Waning immunity and microbial vaccines — workshop of the National Institute of Allergy and Infectious Diseases. Clin Vaccine Immunol. 2017;24:7. doi:10.1128/cvi.00034-17. PMC5498725 28490424

[42] Vesikari T, Ostergaard L, Beeeslaar J, Absalon J, Eiden JJ, Jansen KU, Jones TR, Harris SL, Maansson R, Munson S, et al Persistence and 4-year boosting of the bactericidal response elicited by two- and three-dose schedules of MenB-FHbp: A phase 3 extension study in adolescents. Vaccine. 2019;37:1710–9. doi:10.1016/j.vaccine. 30770221

[43] Reiner DM, Bhuyan P, Eiden JJ, Ginis J, Harris S, Jansen KU, Jiang Q, Jones TR, O’Neill RE, York LJ, et al Immunogenicity, safety, and tolerability of the meningococcal serogroup B bivalent rLP2086 vaccine in adult laboratory workers. Vaccine. 2016;34(6):809–13. doi:10.1016/j.vaccine.2015.12.016. 26707218

[44] Harris SL, Donald RG, Hawkins JC, Tan C, O’Neill R, McNeil LK, Perez JL, Anderson AS, Jansen KU, Jones TR *Neisseria meningitidis* serogroup B vaccine, bivalent rLP2086, induces broad serum bactericidal activity against diverse invasive disease strains including outbreak strains. Pediatr Infect Dis J. 2017;36(2):216–23. doi:10.1097/INF.0000000000001399. 27846061

[45] Taha MK, Hawkins JC, Liberator P, Deghmane AE, Andrew L, Hao L, Jones TR, McNeil LK, O’Neill RE, Perez JL, et al Bactericidal activity of sera from adolescents vaccinated with bivalent rLP2086 against meningococcal serogroup B outbreak strains from France. Vaccine. 2017;35(11):1530–37. doi:10.1016/j.vaccine.2017.01.066. 28196734

[46] Lujan E, Partidge E, Giuntini S, Ram S, Granoff DM Breadth and duration of meningococcal serum bactericidal activity in health care workers and microbiologists immunized with the MenB-FHbp vaccine. Clin Vaccine Immunol. 2017;24:e00121–00117. doi:10.1128/CVI.00121-17. 28566335 PMC5583465

[47] Wang X, Cohn A, Comanducci M, Andrew L, Zhao X, MacNeil JR, Schmink S, Muzzi A, Bambini S, Rappuoli R, et al Prevalence and genetic diversity of candidate vaccine antigens among invasive *Neisseria meningitidis* isolates in the United States. Vaccine. 2011;29(29–30):4739–44. doi:10.1016/j.vaccine.2011.04.092. 21571026

[48] Rajam G, Stella M, Kim E, Paulos S, Boccadifuoco G, Serino L, Carlone G, Medini D Meningococcal antigen typing system (MATS)-based *Neisseria meningitidis* serogroup B coverage prediction for the MenB-4C vaccine in the United States. mSphere. 2017;2(6). doi:10.1128/mSphere.00261-17. PMC5687916 29152576

[49] Bexsero (meningococcal group B vaccine) Full prescribing information. Sovicille (SI) (Italy): GSK Vaccines, Srl; 2018.

[50] Perrett KP, McVernon J, Richmond PC, Marshall H, Nissen M, August A, Percell S, Toneatto D, Nolan T Immune responses to a recombinant, four-component, meningococcal serogroup B vaccine (4CMenB) in adolescents: a phase III, randomized, multicentre, lot-to-lot consistency study. Vaccine. 2015;33(39):5217–24. doi:10.1016/j.vaccine.2015.06.103. 26232542

[51] Block SL, Szenborn L, Daly W, Jackowska T, D’Agostino D, Han L, Dull PM, Smolenov I A comparative evaluation of two investigational meningococcal ABCWY vaccine formulations: results of a phase 2 randomized, controlled trial. Vaccine. 2015;33(21):2500–10. doi:10.1016/j.vaccine.2015.03.001. 25795256

[52] Santolaya ME, O’Ryan ML, Valenzuela MT, Prado V, Vergara R, Munoz A, Toneatto D, Grana G, Wang H, Clemens R, et al Immunogenicity and tolerability of a multicomponent meningococcal serogroup B (4CMenB) vaccine in healthy adolescents in Chile: a phase 2b/3 randomised, observer-blind, placebo-controlled study. Lancet. 2012;379(9816):617–24. doi:10.1016/S0140-6736(11)61713-3. 22260988

[53] Banzhoff A Multicomponent meningococcal B vaccination (4CMenB) of adolescents and college students in the United States. Ther Adv Vaccines. 2017;5(1):3–14. doi:10.1177/2051013616681365. 28344804 PMC5349334

[54] Basta NE, Mahmoud AA, Wolfson J, Ploss A, Heller BL, Hanna S, Johnsen P, Izzo R, Grenfell BT, Findlow J, et al Immunogenicity of a meningococcal B vaccine during a university outbreak. N Engl J Med. 2016;375(3):220–28. doi:10.1056/NEJMoa1514866. 27468058 PMC4992664

[55] Basta N, Wolfson J, Mahmoud A, Heller B, Ploss A, Halloran ME, Bai X, Findlow H, Borrow B 4CMenB vaccine immunogenicity up to 1 year after vaccination among university students. Paper presented at: International Pathogenic Neisseria Conference; 2018; Asilomar, CA, USA.

[56] Parikh SR, Andrews NJ, Beebeejaun K, Campbell H, Ribeiro S, Ward C, White JM, Borrow R, Ramsay ME, Ladhani SN Effectiveness and impact of a reduced infant schedule of 4CMenB vaccine against group B meningococcal disease in England: a national observational cohort study. Lancet. 2016;388(10061):2775–82. doi:10.1016/S0140-6736(16)31921-3. 28100432

[57] Rand CM, Goldstein NPN Patterns of primary care physician visits for US adolescents in 2014: implications for vaccination. Acad Pediatr. 2018;18(2s):S72–S78. doi:10.1016/j.acap.2018.01.002. 29502641

[58] C.S. Mott Children’s Hospital, National Poll on Children’s Health Parents not keeping up with teen vaccines [accessed 2018 926]. http://mottpoll.org/reports-surveys/parents-not-keeping-teen-vaccines.

[59] Kempe A, Allison MA, MacNeil JR, O’Leary ST, Crane LA, Beaty BL, Hurley LP, Brtnikova M, Lindley MC, Liang JL, et al Knowledge and attitudes regarding category B ACIP recommendations among primary care providers for children. Acad Pediatr. 2018;18(7):763–68. doi:10.1016/j.acap.2018.04.005. 29678594 PMC6123258

[60] Marshall GS, Tan L Understanding the category B recommendation for serogroup B meningococcal vaccine. Pediatrics. 2017;139(5):e20163484. doi:10.1542/peds.2016-3484. 28557743

[61] Kempe A, Allison MA, MacNeil JR, O’Leary ST, Crane LA, Beaty BL, Hurley LP, Brtnikova M, Lindley MC, Albert AP Adoption of serogroup B meningococcal vaccine recommendations. Pediatrics. 2018;142(3):e20180344. doi:10.1542/peds.2018-0344. 30126935 PMC6200322

[62] Brady MT Strength and clarity of vaccine recommendations influence providers’ practice. Pediatrics. 2018;142(3):e20181633. doi:10.1542/peds.2018-1633. 30126936

[63] Huang L, Goren A, Lee L, Dempsey A, Srivastava A Disparities in Healthcare Providers’ Interpretation and Implementation of ACIP’s Meningococcal Vaccine Recommendations. Paper presented at: IDWeek; 2018; San Francisco, CA. 10.1080/21645515.2019.1682845 PMC7227692 31634035

[64] Walker TY, Elam-Evans LD, Yankey D, Markowitz LE, Williams CL, Mbaeyi SA, Fredua B, Stokley S National, regional, state, and selected local area vaccination coverage among adolescents aged 13-17 years - United States, 2017. MMWR Morb Mortal Wkly Rep. 2018;67(33):909–17. doi:10.15585/mmwr.mm6733a1. 30138305 PMC6107323

[65] Centers for Disease Control and Prevention Recommended immunization schedule for children and adolescents aged 18 years or younger, United States, 2018 [accessed 2018 926]. https://www.cdc.gov/vaccines/schedules/hcp/imz/child-adolescent.html.

[66] Adolescent Immunization Initiative Rationale for an immunization platform at 16 years of age [accessed 2018 614]. https://www.give2mcv4.org/content/uploads/2017/03/rationale-for-16-year-old-immunization-platform.pdf.

[67] Atkinson W, Immunization Action Coalition Adolescent immunization update and the 16-year-old platform [accessed 2018 926]. www.immunize.org/catg.d/S8005.pdf.

[68] Society for Adolescent Health and Medicine Establishing an immunization platform for 16-year-olds in the United States. J Adolesc Health. 2017;60(4):475–76. doi:10.1016/j.jadohealth.2017.01.011. 28340872

[69] Centers for Disease Control and Prevention Recommended immunization schedule for children and adolescents aged 18 years or younger, United States, 2017 [accessed 2018 19]. https://www.cdc.gov/vaccines/schedules/hcp/imz/child-adolescent.html.

